# The Predictability of Cystatin C for Peripheral Arterial Disease in Chinese Population with Type 2 Diabetes Mellitus

**DOI:** 10.1155/2022/5064264

**Published:** 2022-03-29

**Authors:** Luna Liu, Hai Wang, Jing Ning, Junming Han, Chunxiao Yu, Qingbo Guan

**Affiliations:** ^1^Department of Endocrinology, Shandong Provincial Hospital, Cheeloo College of Medicine, Shandong University, Jinan, Shandong 250021, China; ^2^Shandong Provincial Key Laboratory of Endocrinology and Lipid Metabolism, Institute of Endocrinology and Metabolism, Shandong Academy of Clinical Medicine, Jinan, Shandong 250021, China; ^3^Department of Endocrinology, Shandong Provincial Hospital Affiliated to Shandong First Medical University, Jinan, Shandong 250021, China

## Abstract

**Objectives:**

Peripheral artery disease (PAD) in diabetic populations is a vital chronic disease all over the world due to its high morbidity and mortality. It is important to find early simple screening biomarkers and find residual risk factors that may provide a new target for prevention and treatment of PAD in diabetic patients besides traditional cardiometabolic risk factors.

**Methods:**

We performed a cross-sectional retrospective study, and a total of 1671 T2DM participants were recruited. Receiver operating characteristic analysis, stepwise logistic regression analysis, points score system, and decision curve analysis were performed to assess the risk factors for PAD.

**Results:**

The prevalence of PAD in the study was 7.18% (*n* = 120). Compared to the participants with the lowest quartile of cystatin C (CysC), the risk of developing PAD in participants with the highest quartile of CysC increased 6.339-fold. The CysC was the superior indicators to distinguish participants with PAD from those without PAD, with an AUC of 0.716. Stepwise logistic regression analysis showed that CysC was independent risk factor for PAD besides traditional risk factors. Combined exposure to these traditional risk factors and CysC was associated with a stepwise increase in the risk of developing PAD and even increased 11.976-fold in participants with the highest quintiles of combined exposure score (CES) based on traditional risk factors and CysC compared to the participants with the lowest quintiles of CES.

**Conclusions:**

CysC was associated with PAD independent of potential risk factors in diabetic populations. The CysC was a reliable marker for the early screening of PAD in diabetic patients besides traditional cardiometabolic risk factors.

## 1. Introduction

Peripheral artery disease (PAD) refers to atherosclerotic occlusive disease of the peripheral arteries, which affects more than 100 million people worldwide and imposes a substantial medical and economic burden on patients and society [1]. PAD is also associated with infection, amputation, cardiovascular events and became an increased risk of premature mortality [2, 3]. Diabetes mellitus (DM) has been shown to become one of the strongest risk factors for PAD. Diabetic patients have a higher risk of developing PAD and also have a worse outcome including amputation and mortality than nondiabetic people [4, 5]. Although extensive treatment of classical risk factors for PAD, including blood glucose control and smoking cessation, a high residual risk for PAD in populations with type 2 diabetes mellitus (T2DM) remains. Therefore, it is vital to find residual risk factors that may provide a new target for the prevention and treatment of PAD in diabetic patients. Meanwhile, many patients with PAD were underdiagnosed and undertreated due to the lack of typical symptoms of intermittent claudication, especially in the diabetic participants with neuropathy compliments. Thus, it is necessary to find a simple and inexpensive biomarker for the early and accurate screening of PAD in diabetic populations.

Impaired kidney function is one of the important complications of diabetes that markedly increases the morbidity of cardiovascular disease and mortality. Several studies have shown a negative association between kidney function defined by different indices and PAD in the general population [6–8]. In recent years, the relationship between renal function and PAD in the T2DM population has been paid more attention by scholars due to the coexistence of diabetic nephropathy and diabetic angiopathy. Hsieh et al. showed that diabetic nephropathy evaluated by serum creatinine, creatinine-based estimated glomerular filtration rate (eGFR), and urinary albumin-creatinine ratio (ACR) was significantly associated with PAD, but it did not adjust for smoking status which is considered as the most important risk factor for PAD [9]. Another study based on 478 patients with T2DM aged more than 50 years old demonstrated that macroalbuminuria was a stronger indicator for PAD than eGFR < 60 ml/min/1.73 m^2^ [10]. A recent study showed that cystatin C (CysC) was independently associated with PAD in type 2 diabetes mellitus patients without overt nephropathy, while 24 h-urine albumin and serum creatine were not independent risk factors for PAD [11]. However, there is no study to systematically assess the relationship of all renal function parameters and PAD in a large-scale T2DM population and investigate which indicator was independently associated with PAD and became the most potent predictive indicator for developing PAD in population with T2DM.

The biomarkers that can assess the renal function in T2DM patients are diverse and complicated, but whether these indicators can become one of the potent predictive indicators for PAD remains unknown. The eGFR is recognized as the clinical standard measurement to assess kidney function. However, a lower generation of creatinine because of the loss of muscle in PAD patients makes it inaccurate when we calculate the eGFR based on creatine which is the byproduct of muscle breakdown [12]. Apart from eGFR and creatinine, albuminuria has long been identified as the major screening tool to detect kidney complications in patients with T2DM, but many diabetic patients with impaired kidney function have the normal range of albuminuria [13]. The CysC, beta 2 microglobulins (BMG), and retinol binding protein (RBP) have been considered as alternative markers for the detection of impaired renal function. And these markers are produced at a relatively stable state and could not be affected by muscle breakdown [14, 15]. Therefore, it is needed to verify the relationship between these indicators and PAD and further investigate whether the combination of renal function indicator and conventional risk factors such as age, diabetic course, and smoking status can increase the predictive power for developing PAD in population with T2DM.

Thus, we performed this study to evaluate the independent association between renal function parameters and PAD in Chinese populations with type 2 diabetes mellitus and compare the prediction power of renal function parameters including eGFR, creatinine, CysC, BMG, and RBP for early screening of individuals with elevated risk for PAD and identify a cutoff for the diagnosis of PAD. We also investigate whether a combination of renal function parameters and traditional potential risk factors could increase the predictability of PAD than simple traditional risk factors.

## 2. Subjects and Methods

### 2.1. Subjects

We retrospectively obtained the medical records of patients with T2DM who were admitted into Shandong Provincial Hospital between Aug 2017 and Oct 2020. In our study, the exclusion criteria were as follows: (i) type I diabetes mellitus and other specific types of diabetes mellitus; (ii) no information on vital statistics (such as age, sex, and BMI) or missing data on ABI, renal function, serum glucose levels, and lipid profiles; and (iii) severe hepatic or renal disorders, lung diseases, hypothalamus and/or pituitary gland diseases, neurologic diseases, or tumors. At last, a total of 1671 participants were recruited in this study (Sup Figure 1). All participants gave informed consent by telephone. This study was approved by the Ethics Committee of Shandong Provincial Hospital, and the records were used for research purposes only.

### 2.2. Data and Specimen Collection

The baseline demographic information was collected from the medical records of each eligible participant, which was consisted of sex, age, height, weight, diabetes course, and lipid profiles including total cholesterol (TC), triglyceride (TG), high-density lipoprotein cholesterol (HDL-C), low-density lipoprotein cholesterol (LDL-C), fasting blood glucose levels (FPG), glycosylated hemoglobin (HbA1c), C-P, insulin, uric acid and homocysteine (HCY), and renal function indices including urea nitrogen (BUN), creatinine (CREA), RBP, BMG and CysC, liver function indices, blood cell count, coagulation indices, C reactive protein (CRP), ABI, urine microalbuminuria (MALB), urine creatine (uCREA), and past medical history (including hypertension, coronary heart disease, and cerebrovascular disease). Indicators of the first test were included in the analysis if the patients had repeated laboratory tests given that the first test can better reflect the body status and eliminate the influence of medications and treatments during hospitalization. Height and weight were measured adjusting by 0.1 cm and 0.1 kg, respectively. BMI was calculated by dividing weight in kilograms by the square of height in meters. Smoking status and alcohol consumption were considered as never, ever, and current.

### 2.3. PAD Evaluation

ABI is an objective diagnostic method to detect PAD. ABI of each eligible participant was measured during the hospitalization by arteriosclerosis detector (bp-203rpe, Omron) in Shandong Provincial Hospital. Systolic blood pressure was measured of each brachial artery and posterior tibial artery. The ABI of the left and the right side was calculated by dividing the systolic pressure of each leg by each brachial artery systolic pressure, respectively. PAD was defined as ABI ≤ 0.90 on either side [16], and non-PAD was defined as ABI from 0.90 to 1.40. When patients had ABI > 1.40, they were excluded because of the inability to evaluate the vascular perfusion adequately.

### 2.4. Statistical Analysis

Continuous variables were presented as mean ± standard deviations (SD), and between-group differences were assessed by an independent two-sample *t*-test. Categorical variables were presented as percentages, and between-group differences were assessed by chi-squared test. We used complete data for further analysis due to the low missing rate and random missing and processed continuous variables into classified variables for subsequent analysis to avoid bias caused by outliners. Univariate regression and stepwise logistic regression analysis were performed to assess the risk factors for PAD. We chose an entry probability of <0.05 by the stepwise selection method. Receiver operating characteristic (ROC) analyses were performed to identify a diagnostic cut-off and compare the predictive efficiency of different risk factors. To investigate the combined effect of traditional risk factors and renal function parameters, we calculated the combined exposure score of each participant based on the regression coefficient in the stepwise logistic regression model based on the previous points system method [17, 18]. We defined the regression coefficient of age as the constant, divided the regression coefficient of each risk factor to the selected constant, and rounded to the nearest integer to obtain the risk point of each risk factor. The combined exposure score of each participant was derived by summing the risk points of each risk factor. Then, the combined exposure score was categorized into quintiles, and logistic regression analysis was performed to compare the risk of developing PAD across them. Simultaneously, we contrasted two predictive models: conventional model based on traditional risk factors and complex model based on traditional risk factors and CysC. Decision curve analysis (DCA) was performed to compare the net benefit of two models for predicting PAD. All statistical analyses were performed using SPSS statistical software (version22.0) and *R* software. Statistical significance was considered as a *p* value <0.05.

## 3. Results

### 3.1. Baseline Characteristics of the Studied Population

A total of 1671 participants with T2DM were enrolled into the study, and 7.18% were diagnosed with PAD according to ABI values (*n* = 120). Participants with PAD were likely to have an older age, longer diabetic course, higher HbA1C, HCY, CREA, CsyC, WBC, PLT, and Fib levels and lower ALT and RBC levels (Table 1). No significant difference existed in the BMI, C-P, FPG, insulin, TC, TG, LDL-C, HDL-C, AST, URIC, and CRP levels between the participants with and without PAD. There is a higher proportion of hypertension, cardiovascular disease, cerebrovascular disease, current smoking, and a lower percentage of current drinking in participants with PAD compared to individuals without PAD (Table 1). Individuals with PAD are likely to have an increased eGFR, CysC, BMG, RBP, uMALB, and uMALB/uCREA (ACR) levels in comparison to those without PAD (Table 1). The results about the interaction between variables and multicollinearity were shown in Sup Tab 1 and Sup Tab 2 (Sup Tab).

### 3.2. Univariate Logistic Regression Analysis between Risk Factors and PAD

To investigate the association of potential risk factors and PAD, we performed univariate logistic regression analysis in the studied populations with T2DM. The odd ratios for PAD in participants with the highest quartiles of age, diabetes course, HbA1C, HCY, WBC, PLT, Fib, URIC, BUN, and BMG levels in contrast to the lowest quartiles were 11.237, 5.346, 1.928, 2.825, 2.065, 1.640, 4.531, 1.461, 3.099, and 6.919, respectively (Table 2). Compared to the participants with the lowest quartile of cystatin C (CysC), the risk of developing PAD in participants with the highest quartile of CysC increased 6.339-fold (95% CI: 3.279-12.254) (Table 2).

### 3.3. Receiver Operating Characteristic Analysis of Potential Risk Factors to Predict PAD

**Figure 1:**
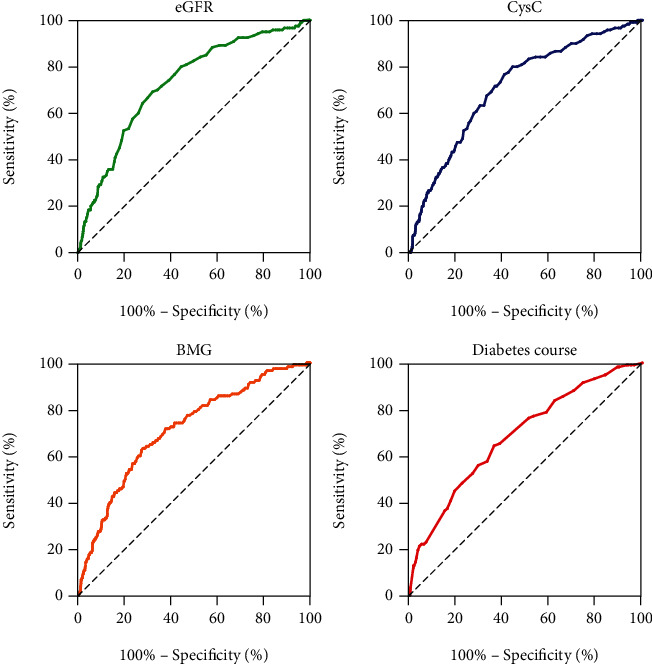
ROC curve of potential risk factors to predict PAD. Abbreviations: ROC: receiver operating characteristic; PAD: peripheral artery disease; eGFR: estimated glomerular filtration rate; CysC: cystatin C; BMG: beta 2 microglobulin.

We used receiver operating characteristic (ROC) analysis to compare the prediction power of potential risk factors for PAD (Figure 1). Among all the predicting indexes, eGFR, CysC, and BMG were the superior indicators to distinguish participants with PAD from those without PAD, with area under curves (AUC) of 0.728, 0.716, and 0.713, respectively, even higher than diabetes course (Figure 1). The optimal cutoff point of eGFR for PAD was 95.50 ml/min. The sensitivity and specificity at this level were 69.2% and 67.8%, respectively (Table 3), while the optimal cutoff points of CysC and BMG for PAD were 0.915 mg/L and 2.240 mg/L, respectively (Table 3). The sensitivity and specificity at this level were 76.7% and 59.4% for CysC and 63.3% and 72.0% for BMG (Table 3). Other details of all the risk factors for the PAD including diabetic course, WBC, RBC, PLT, BUN, CREA, and RBP were also reported in this study (Table 3, Sup Figure 2).

### 3.4. Stepwise Logistic Regression Analysis of Risk Factors for PAD

To study the independent effect of renal function parameters on PAD, we performed stepwise logistic regression analysis in the populations with T2DM, respectively. According to the cut-off obtained from ROC analysis (Table 3), we defined the cut point level of each continuous variable and divided the renal function parameters into higher and lower groups. The risk factors used in stepwise logistic regression analysis included age, sex, CysC, PLT, WBC, RBC, diabetes course, smoking status, BUN, BMG, eGFR, HCY, Fib, and ALT. We chose an entry probability of <0.05 by the stepwise selection method. As it is shown in Table 4, age, sex, CysC, PLT, WBC, RBC, diabetes course, and smoking status were independently associated with the risk of PAD (Table 4). The odds ratios (ORs) for the presence of PAD in the higher group of age, sex, CysC, PLT, WBC, RBC, diabetes course, and smoking status were 1.075 (95% CI, 1.049-1.102), 2.866 (95% CI, 1.571-5.227), 2.497 (95% CI, 1.535-4.064), 1.771 (95% CI, 1.097-2.859), 2.856 (95% CI, 1.673-4.873), 2.090 (95% CI, 1.334-3.276), 1.667 (95% CI, 1.078-2.577), and 6.040 (95% CI, 3.126-11.672), respectively (Table 4).

### 3.5. Combined Effects of Traditional Risk Factors and CysC Levels for Predicting PAD

**Figure 2:**
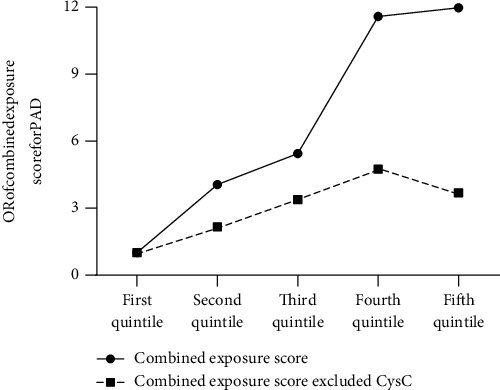
The odds ratio of developing PAD among the quintiles of the combined exposure score. The odds ratio of developing PAD among the quintiles of the combined exposure score. Combined exposure score was obtained based on risk points of age, sex, smoking status, diabetic course, platelets (PLT), red blood cell (RBC), white blood cell (WBC), and cystatin C (CysC). Combined exposure score excluded CysC was calculated based on risk points of age, sex, smoking status, diabetic course, PLT, RBC, and WBC.

**Figure 3:**
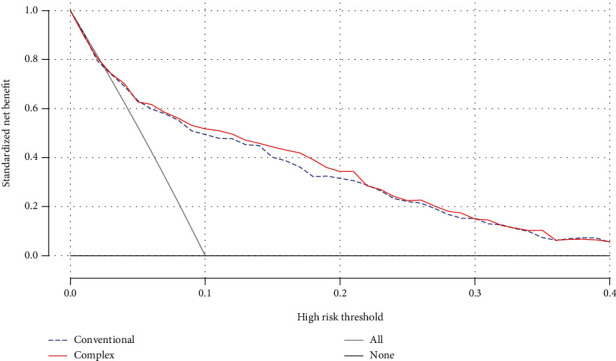
Decision curve analysis of the two prediction models. *X*-axis indicates the threshold probability for critical care outcome, and *Y*-axis indicates the net benefit. Blue line = conventional model based on age, sex, smoking status, diabetic course, platelets (PLT), red blood cell (RBC), and white blood cell (WBC) and red line = complex model based on age, sex, smoking status, diabetic course, PLT, RBC, WBC, and cystatin C.

To further investigate whether a combination of CysC and traditional risk factors could increase the predictability of PAD than simple traditional risk factors, we used points score system and decision curve analysis (DCA). As shown in Table 4, age, sex, RBC, WBC, PLT, smoking status, diabetes course, and CysC were significantly associated with the risk of developing PAD, except for other renal function parameters in the stepwise logistic regression analysis model (Table 4). To further investigate the diagnostic power of the combined effects of risk factors for PAD, we calculated the risk points of each significantly related risk factor which equaled the risk of developing PAD with each year increase in age in this population (Table 4). The risk point was obtained by dividing the regression coefficient of each risk factor by the regression coefficient of age [17, 18]. We divided the combined exposure score derived by summing the individual risk points for each participant into quintiles. Compared to the lowest quintiles of combined exposure score based on sex, RBC, WBC, PLT, diabetes course, and smoking status, the participants in the highest quintiles were 3.686-fold more likely to develop PAD in the T2DM population (Figure 2). Combined exposure to these risk factors including traditional risk factors and CysC was associated with a stepwise increase in the risk of developing PAD. The risk of developing PAD in the participants with the highest quintiles of combined exposure score based on CysC, sex, RBC, WBC, PLT, diabetes course, and smoking status increased 11.976-fold in contrast to the lowest quintiles (Figure 2). DCA analysis also showed that the net benefit of the complex model based on CysC and age, sex, RBC, WBC, PLT, diabetes course, and smoking status was larger than the range of conventional risk model, which indicated that complex model was superior for predicting PAD than simple conventional model in populations with T2DM (Figure 3).

## 4. Discussion

Peripheral artery disease in diabetic populations is a vital chronic disease all over the world due to its high morbidity and mortality. It is important to find residual risk factor that may provide new target for prevention and treatment of PAD in diabetic patients besides traditional risk factors and find simple biomarker to increase early screening rate of PAD in diabetic patients. In the present study, we provided precise insight into the association between renal function parameters and PAD in populations with T2DM. It demonstrated that CysC was an independent risk factor for PAD in populations with T2DM. Moreover, combined effects of the traditional risk factors of PAD and CysC can increase the predictability of PAD than simple traditional risk factor for PAD. These findings suggested that CysC was a reliable marker for the screening of PAD in diabetic patients.

The prevalence of PAD in our studied T2DM population was 7.18%. Previous studies have reported variations in the prevalence of PAD ranging from 6.5% to 32.2% in Chinese T2DM population [13, 19, 20]. Given that our studied participants were higher than 30 years old, which were younger than other studied populations, the prevalence of PAD in our study remained at a relatively low level. In the clinical setting, many of these patients were underdiagnosed and undertreated due to the lack of typical symptom of intermittent claudication, especially in the diabetic participants with neuropathy compliments. Angiography, recognized as the gold standard method for diagnosis of PAD, is unsuitable for screening due to its invasiveness and high price. Alternatively, the measurement of ABI is a noninvasive and relatively inexpensive technique employed in the evaluation of PAD based on studies reporting >90% sensitivity and specificity compared with angiography [16]. However, many hospitals especially basic-level hospitals lack specialized equipment and trained personnel to perform ABI measurements in China. Therefore, it is necessary to find a method that is simple and inexpensive for the early screening of PAD in the T2DM populations.

In the present study, we firstly verified the traditional risk factors for PAD in participants with T2DM. As the results showed, participants with PAD were more likely to be current smoker, which was consistent with the previous study [21]. More females than males had PAD in the study, which is in line with the previous study [22]. This was likely due to the worse metabolic factor and poor glycemic control in the female diabetic participants especially postmenopausal women. However, it is inconsistent with the previous notion that PAD was more common in men due to the higher proportion of smokers [23]. However, we did not find the association between lipid profiles and PAD in the participants, which was inconsistent with previous study [24]. It is our theory that in the face of multiple risk factors for PAD including aging, smoking and persistent hyperglycemia, the role of dyslipidemia may be less substantial. In our study, we showed that a longer duration of DM and poor long-term glycemic was significantly associated with the prevalence of PAD in the studied diabetic population. It also demonstrates that it is vital to properly control glucose levels and pay attention to early diagnosis and treatment of PAD in the T2DM population. Apart from these traditional risk factors, we focus on the association between renal function parameters and PAD to provide new target for diagnosis and treatment of PAD in the T2DM population in the present study.

In our study, we demonstrated that renal function index especially CysC was the independent risk factor for PAD in populations with T2DM. A potential explanation of the relationship between diabetic nephropathy and PAD was the coprogression of renal artery stenosis and overall atherosclerosis including peripheral arterial disease [25]. We also found that patients with PAD had a higher percentage of developing cardiovascular and cerebrovascular disease, which indicated the coexist of overall atherosclerosis in the T2DM patients. However, we did not get information about diabetic retinopathy in the studied population. Another possible mechanism underlying this relationship was inflammation pathways which were considered as the prominent component of atherosclerosis. It has been reported to play central roles in the progression of diabetic nephropathy [26]. The previous study has showed that inflammatory modulator CRP was predictive of the development of PAD in the general population [27]. In our study, we found that inflammatory indicator WBC rather than CRP was positively correlated with the prevalence of PAD. Besides the inflammatory pathways, coagulation disturbance which was associated with an increased risk of developing PAD [28], played pathogenic roles in the relationship between diabetic nephropathy and high prevalence of atherosclerotic cardiovascular disease [29]. Therefore, we hypothesized that coagulation disturbance in the patients with diabetic nephropathy would contribute to the development and progression of PAD. However, after adjusting for these potential risk factors including inflammation and coagulation parameters, the inverse association between diabetic nephropathy and prevalence of PAD still existed in our study, which indicated that other mechanisms were involved in the emergence of diabetic nephropathy as an independent risk factor for PAD in population with T2DM.

The relationship between renal function parameters and PAD has been previously studied based on clinical studies, but there are various indexes used for evaluating renal function including albuminuria and infiltration indicators. No study has been performed to comprehensively examine the relationship between various renal function indexes and PAD, and the further comparison of the discriminability of most renal function parameters for the risk of PAD has not been reported. In our study, we demonstrated that CysC, BMG, and eGFR were the potent predictive indicator for the PAD via ROC analysis. In the stepwise logistic regression model, only CysC was significantly associated with the risk of PAD. We also found that combined effects of the traditional risk factors of PAD and CysC can increase the predictability of PAD than simple traditional risk factor for PAD via point score system and decision curve analysis. It all indicated that CysC was a potent predictive measurement for the PAD in the T2DM population. Previous studies have demonstrated that CysC, considered as the filtration marker of renal function, was associated with the prevalence of PAD in the general population [30, 31]. And CysC is produced at a relatively stable state and could not be affected by muscle breakdown [8], which can partly explain the potent association between CysC and PAD. Apart from renal function, the previous study has reported the association between CysC and cardiovascular events, which can also increase evidence for the independent association of CysC and PAD [32]. Our study firstly reported the association between BMG and PAD in the diabetic population, and the predictive power of BMG for PAD was also potent although slightly weaker than CysC. And our study has firstly involved the relationship between RBP and PAD. Although RBP was a suitable indicator for renal function, it was not a suitable biomarker for predicting the prevalence of PAD.

To the best of our knowledge, this is the first study to point out that CysC was a reliable predictive marker for the screening of PAD in type 2 diabetes mellitus patients. However, several limitations also existed in this study. The cross-sectional study was unable to detect any causal relationship between renal function parameters and PAD. PAD was diagnosed based on the ABI measurement instead of angiography in our study, which is the gold standard method for diagnosis. And it has to be stressed that the number of our studied population was not too high, and participants with PAD were even lower in the studied population. Thus, the power to conclude the association between renal function parameters and PAD may not be sufficient. Large prospective studies are needed to validate this relationship.

## 5. Conclusions

In conclusion, we demonstrated that CysC was the independent risk factor for developing PAD in populations with T2DM, and we also found that CysC was a reliable marker for the screening of PAD in type 2 diabetes mellitus patients. The findings on CysC and PAD risk may have applications in the clinical setting because determination of renal function parameters is already widely available and routinely measured in clinical settings. Clinical physicians should pay attention to the improvement of vascular ultrasound and ABI examination in diabetic patients with CysC higher than 0.915 mg/L. And the exploration of the possible mechanisms underlying renal function parameters and PAD may provide more approaches to prevent and treat PAD in the future.

## Figures and Tables

**Table 1:** Baseline characteristics of studied population.

Characteristics	Non-PAD (*n* = 1551)	PAD (*n* = 120)	*p*
Age (years)	57.56 ± 11.83	68.04 ± 9.83	<0.001
Sex (male %)	56.3	36.7	<0.001
BMI (kg/m^2^)	25.60 ± 3.73	25.03 ± 4.17	0.114
Diabetes course (years)	9.84 ± 7.38	15.70 ± 9.11	<0.001
FPG (mmol/L)	8.37 ± 2.85	8.62 ± 3.53	0.457
HbA1C (%)	8.85 ± 1.96	9.25 ± 2.10	0.034
Insulin (uIU/ml)	13.77 ± 36.59	20.87 ± 46.20	0.120
C-P (ng/ml)	1.95 ± 1.16	1.96 ± 1.42	0.974
TG (mmol/L)	1.76 ± 1.57	1.81 ± 1.30	0.741
TC (mmol/L)	4.97 ± 1.34	4.99 ± 1.23	0.891
LDL-C (mmol/L)	3.08 ± 0.95	3.07 ± 0.96	0.853
HDL-C (mmol/L)	1.19 ± 0.32	1.18 ± 0.28	0.782
HCY (umol/L)	12.25 ± 6.73	14.07 ± 9.22	0.006
ALT (U/L)	23.09 ± 20.29	16.99 ± 9.50	<0.001
AST (U/L)	22.88 ± 15.92	19.83 ± 6.23	<0.001
RBC (10^12^/L)	4.65 ± 0.57	4.34 ± 0.60	<0.001
WBC (10^9^/L)	6.46 ± 1.96	7.07 ± 2.55	0.011
PLT (10^9^/L)	230.89 ± 64.83	249.32 ± 83.03	0.003
Fib (g/l)	3.10 ± 0.94	3.61 ± 1.16	<0.001
CRP (mg/L)	6.23 ± 22.22	9.45 ± 27.70	0.207
URIC (umol/L)	330.71 ± 207.79	401.63 ± 577.26	0.183
BUN (mmol/L)	5.53 ± 3.17	6.46 ± 2.83	0.001
CREA (umol/L)	68.33 ± 145.68	72.94 ± 33.70	0.730
eGFR	100.35 ± 20.33	84.02 ± 22.82	<0.001
BMG (mg/L)	2.12 ± 1.13	2.83 ± 1.31	<0.001
CysC (mg/L)	0.94 ± 0.36	1.21 ± 0.48	<0.001
RBP (mg/L)	42.46 ± 13.76	44.60 ± 15.68	0.106
uMALB (mg/L)	93.50 ± 357.50	220.00 ± 633.11	0.033
uCREA (mmol/L)	7.88 ± 4.87	5.53 ± 3.71	<0.001
uMALB/uCREA (mg/g)	197.91 ± 969.01	483.03 ± 1186.63	0.013
Hypertension (%)	49.5	75.4	<0.001
CHD (%)	22.5	42.4	<0.001
CVD (%)	11.7	31.4	<0.001
Smoking status (%)			0.048
Never	64.4	62.7	
Ever	14.6	8.5	
Current	21.0	28.8	
Drinking status (%)			0.003
Never	60.8	76.3	
Ever	10.2	8.5	
Current	29.0	15.3	

Abbreviations: PAD: peripheral artery disease; BMI: body mass index; FPG: fasting blood glucose levels; HbA1C: glycosylated hemoglobin; TG: triglyceride; TC: total cholesterol; LDL-C: low-density lipoprotein cholesterol; HDL-C: high-density lipoprotein cholesterol; HCY: homocysteine; ALT: alanine aminotransferase; AST: aspartate aminotransferase; RBC: red blood cell; WBC: white blood cell; PLT: platelets; Fib: fibrinogen; CRP: C reactive protein; URIC: uric acid; BUN: urea nitrogen; CREA: creatinine; eGFR: estimated glomerular filtration rate; BMG: beta 2 microglobulin; CysC: cystatin C; RBP: retinol binding protein; uMALB: urine microalbumin; uCREA: urine creatinine; CHD: coronary heart disease; CVD: cerebrovascular disease.

**Table 2:** Univariate logistic regression analysis between risk factors and PAD.

Characteristics	Q1	Q2	Q3	Q4
Age (years)	Ref.	1.308 (0.526-3.253)	3.649 (1.717-7.757)	11.237 (5.530-22.836)
Diabetes course (years)	Ref.	1.783 (0.923-3.443)	2.599 (1.290-5.235)	5.346 (2.931-9.752)
HbA1C (%)	Ref.	2.311 (1.251-4.272)	2.646 (1.450-4.830)	1.928 (1.026-3.623)
HCY (umol/L)	Ref.	1.141 (0.608-2.140)	1.697 (0.939-3.067)	2.825 (1.633-4.888)
ALT (U/L)	Ref.	0.605 (0.382-0.957)	0.368 (0.214-0.632)	0.285 (0.158-0.514)
RBC (10^12^/L)	Ref.	0.575 (0.367-0.902)	0.290 (0.167-0.503)	0.198 (0.104-0.375)
WBC (10^9^/L)	Ref.	1.590 (0.891-2.839)	1.576 (0.880-2.823)	2.065 (1.183-3.605)
PLT (10^9^/L)	Ref.	1.141 (0.643-2.021)	1.459 (0.847-2.515)	1.640 (0.963-2.794)
Fib (g/L)	Ref.	2.072 (1.022-4.200)	3.142 (1.607-6.143)	4.531 (2.373-8.652)
URIC (umol/L)	Ref.	1.295 (0.746-2.246)	1.346 (0.776-2.336)	1.461 (0.850-2.510)
BUN (mmol/L)	Ref.	1.493 (0.809-2.753)	1.496 (0.807-2.773)	3.099 (1.772-5.419)
CREA (umol/L)	Ref.	1.086 (0.613-1.924)	0.980 (0.544-1.766)	2.194 (1.315-3.660)
eGFR	Ref.	0.390 (0.248-0.612)	0.172 (0.094-0.317)	0.113 (0.053-0.237)
BMG (mg/L)	Ref.	1.469 (0.673-3.204)	2.739 (1.345-5.577)	6.919 (3.595-13.316)
CysC (mg/L)	Ref.	1.168 (0.517-2.636)	3.865 (1.943-7.689)	6.339 (3.279-12.254)
RBP (mg/L)	Ref.	1.125 (0.641-1.973)	1.496 (0.881-2.539)	1.340 (0.780-2.304)
uMALB (mg/L)	Ref.	1.248 (0.649-2.401)	1.448 (0.766-2.738)	3.751 (2.142-6.568)
uCREA (mmol/L)	Ref.	0.597 (0.375-0.951)	0.384 (0.226-0.651)	0.195 (0.100-0.380)
uMALB/uCREA (mg/g)	Ref.	2.715 (1.188-6.204)	3.830 (1.729-8.484)	8.164 (3.841-17.352)

Abbreviations: PAD: peripheral artery disease; *Q*: quartile; Ref.: reference; HbA1C: glycosylated hemoglobin; HCY: homocysteine; ALT: alanine aminotransferase; RBC: red blood cell; WBC: white blood cell; PLT: platelets; Fib: fibrinogen; URIC: uric acid; BUN: urea nitrogen; CREA: creatinine; eGFR: estimated glomerular filtration rate; BMG: beta 2 microglobulin; CysC: cystatin C; RBP: retinol binding protein; uMALB: urine microalbumin; uCREA: urine creatinine.

**Table 3:** AUCs, cut-off, sensitivity, and specificity of risk factors in ROC analysis to predict PAD.

Characteristics	AUC	95% CI	Cut-off	Sensitivity	Specificity	Youden index
eGFR	0.728	0.682-0.773	95.500	0.692	0.678	0.369
CysC (mg/L)	0.716	0.669-0.762	0.915	0.767	0.594	0.360
BMG (mg/L)	0.713	0.665-0.761	2.240	0.633	0.720	0.353
Diabetes course (years)	0.688	0.638-0.738	11.500	0.644	0.638	0.282
RBC (10^12^/L)	0.668	0.619-0.717	4.455	0.617	0.671	0.288
uCREA (mg/L)	0.663	0.614-0.711	5.840	0.704	0.581	0.286
uMALB (mmol/L)	0.649	0.596-0.702	21.380	0.479	0.768	0.247
BUN (mmol/L)	0.616	0.562-0.670	6.550	0.392	0.798	0.190
CREA (umol/L)	0.581	0.523-0.638	73.400	0.383	0.789	0.173
WBC (10^9^/L)	0.574	0.520-0.628	8.975	0.225	0.913	0.138
PLT (10^9^/L)	0.563	0.510-0.616	206.500	0.742	0.371	0.113
RBP (mg/L)	0.541	0.489-0.593	42.350	0.533	0.563	0.097

Abbreviations: AUC: area under curve; ROC: receiver operating characteristic; PAD: peripheral artery disease; 95% CI: 95% credibility interval; eGFR: estimated glomerular filtration rate; CysC: cystatin C; BMG: beta 2 microglobulin; RBC: red blood cell; uCREA: urine creatinine; uMALB: urine microalbumin; BUN: urea nitrogen; CREA: creatinine; WBC: white blood cell; PLT: platelets; RBP: retinol binding protein.

**Table 4:** Main risk factors associated with increased risk of developing PAD.

Risk factor	OR (95% CI)	*p*	Regression coefficient	Risk points
Age	1.075 (1.049-1.102)	<0.001	0.073	1
Smoking status				
Never	1.00			0
Ever	1.233 (0.528-2.881)	0.629	0.209	3
Current	6.040 (3.126-11.672)	<0.001	1.798	25
Sex				
Male	1.00			0
Female	2.866 (1.571-5.227)	0.001	1.053	14
WBC (10^9^/L)				
<8.975	1.00			0
≥8.975	2.856 (1.673-4.873)	<0.001	1.049	14
CysC (mg/L)				
<0.915	1.00			0
≥0.915	2.497 (1.535-4.064)	<0.001	0.915	13
RBC (10^12^/L)				
≥4.455	1.00			0
<4.455	2.090 (1.334-3.276)	0.001	0.737	10
Diabetic course (years)				
<11.5	1.00			0
≥11.5	1.667 (1.078-2.577)	0.021	0.511	7
PLT (10^9^/L)				
<206.500	1.00			0
≥206.500	1.771 (1.097-2.859)	0.019	0.572	8

Main risk factors associated with increased risk of developing PAD. This table used the stepwise logistic regression analysis to find the independent risk factor for developing of PAD. The risk factors used in stepwise logistic regression analysis included age, sex, CysC, PLT, WBC, RBC, diabetes course, smoking status, urea nitrogen, beta 2 microglobulin, estimated glomerular filtration rate, homocysteine, fibrinogen, and alanine aminotransferase. We chose an entry probability of <0.05 by the stepwise selection method. The risk points of each significantly associated risk factors are created by dividing the regression coefficient of each risk factor by the selected constant (regression coefficient of age) and then rounded to the nearest integer. One risk point equals the risk of developing PAD with each year increase in age in this population. To assess the PAD risk associated with combined exposure, a combined exposure score was obtained by summing the individual risk points for each participant. Abbreviations: WBC: white blood cell; CysC: cystatin C; RBC: red blood cell; PLT: platelets.

## Data Availability

The datasets generated during and analyzed during the current study are not publicly available but are available from the corresponding authors on reasonable request.

## Supplementary Materials

Supplementary Materials Sup Table 1: General linear model analysis of potential risk factors for PAD. Abbreviations: PAD: peripheral artery disease; CysC: cystatin C; HBP: hypertension; PLT: platelets. Sup Table 2: multiple linear regression analysis and collinearity diagnostics of potential risk factors for PAD and ABI measurements. Abbreviations: PAD: peripheral artery disease; ABI: ankle-brachial index; RBC: red blood cell; WBC: white blood cell; PLT: platelets; CysC: cystatin C. Sup Figure 1: flowchart of the study. Sup Figure 2: ROC curve of other potential risk factors to predict PAD. Abbreviations: ROC: receiver operating characteristic; PAD: peripheral artery disease; RBC: red blood cell; uCREA: urine creatinine; uMALB: urine microalbumin; BUN: urea nitrogen; CREA: creatinine; WBC: white blood cell; PLT: platelets; RBP: retinol binding protein.
