# Supplementary material for: The Predictability of Cystatin C for Peripheral Arterial Disease in Chinese Population with Type 2 Diabetes Mellitus
Source: J Diabetes Res. 2022 Mar 29;2022:5064264. doi: 10.1155/2022/5064264 (PMC8983175; doi:10.1155/2022/5064264)
Supplement: Supplementary Materials — Sup Table 1: General linear model analysis of potential risk factors for PAD. Abbreviations: PAD: peripheral artery disease; CysC: cystatin C; HBP: hypertension; PLT: platelets. Sup Table 2: multiple linear regression analysis and collinearity diagnostics of potential risk factors for PAD and ABI measurements. Abbreviations: PAD: peripheral artery disease; ABI: ankle-brachial index; RBC: red blood cell; WBC: white blood cell; PLT: platelets; CysC: cystatin C. Sup Figure 1: flowchart of the study. Sup Figure 2: ROC curve of other potential risk factors to predict PAD. Abbreviations: ROC: receiver operating characteristic; PAD: peripheral artery disease; RBC: red blood cell; uCREA: urine creatinine; uMALB: urine microalbumin; BUN: urea nitrogen; CREA: creatinine; WBC: white blood cell; PLT: platelets; RBP: retinol binding protein. [file 5064264.f1.zip › Sup Tab.docx]

Sup Table 1 General linear model analysis of potential risk factors for PAD.

| Factors | Interaction P |
| --- | --- |
| CysC*age | 0.271 |
| CysC*sex | 0.052 |
| CysC*diabetes course | 0.189 |
| CysC* smoking status | 0.120 |
| CysC*HBP history | 0.115 |
| CysC*PLT | 0.663 |

Abbreviations: PAD: peripheral artery disease; CysC: Cystatin C; HBP: hypertension; PLT: platelets.

Sup Table 2 Multiple linear regression analysis and collinearity diagnostics of potential risk factors for PAD and ABI measurements.

| Factors | tolerance | VIF |
| --- | --- | --- |
| age | 0.655 | 1.526 |
| Sex | 0.606 | 1.650 |
| Smoking status | 0.682 | 1.466 |
| Diabetes course | 0.764 | 1.310 |
| RBC | 0.758 | 1.320 |
| WBC | 0.893 | 1.120 |
| PLT | 0.849 | 1.178 |
| CysC | 0.807 | 1.240 |

Abbreviations: PAD: peripheral artery disease; ABI: Ankle-Brachial Index; RBC: red blood cell; WBC: white blood cell; PLT: platelets; CysC: Cystatin C.
