# Supplementary figures and images for: The Predictability of Cystatin C for Peripheral Arterial Disease in Chinese Population with Type 2 Diabetes Mellitus
Source: J Diabetes Res. 2022 Mar 29;2022:5064264. doi: 10.1155/2022/5064264 (PMC8983175; doi:10.1155/2022/5064264)

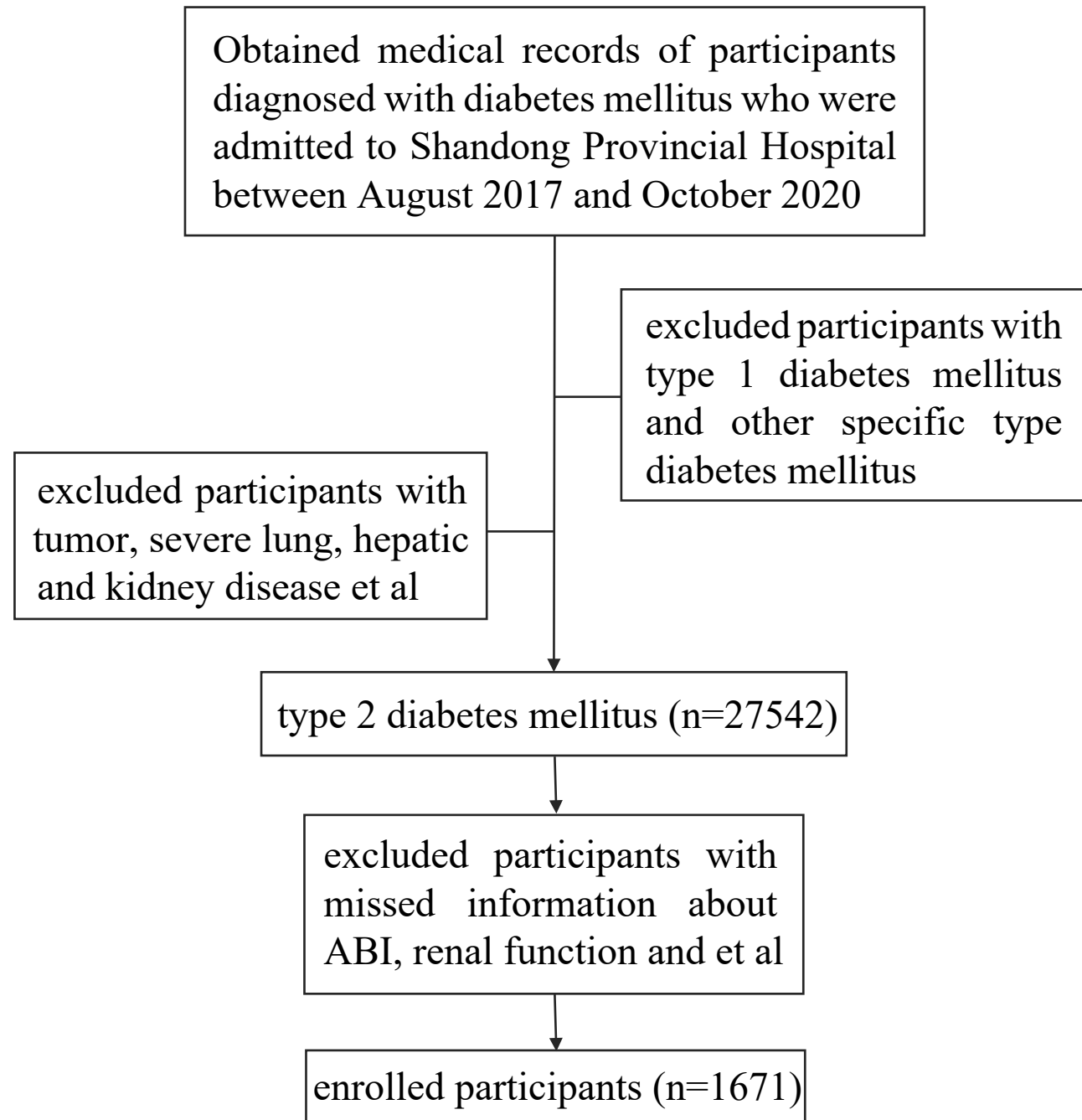

Supplement: Supplementary Materials — Sup Table 1: General linear model analysis of potential risk factors for PAD. Abbreviations: PAD: peripheral artery disease; CysC: cystatin C; HBP: hypertension; PLT: platelets. Sup Table 2: multiple linear regression analysis and collinearity diagnostics of potential risk factors for PAD and ABI measurements. Abbreviations: PAD: peripheral artery disease; ABI: ankle-brachial index; RBC: red blood cell; WBC: white blood cell; PLT: platelets; CysC: cystatin C. Sup Figure 1: flowchart of the study. Sup Figure 2: ROC curve of other potential risk factors to predict PAD. Abbreviations: ROC: receiver operating characteristic; PAD: peripheral artery disease; RBC: red blood cell; uCREA: urine creatinine; uMALB: urine microalbumin; BUN: urea nitrogen; CREA: creatinine; WBC: white blood cell; PLT: platelets; RBP: retinol binding protein. [file 5064264.f1.zip › Sup fig1 (1).pdf]

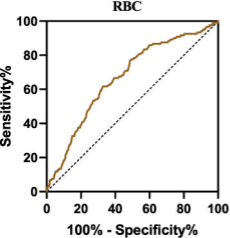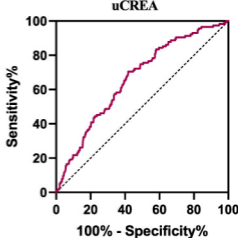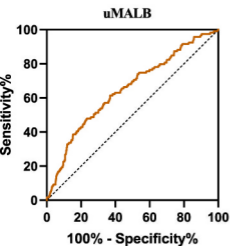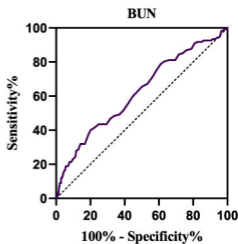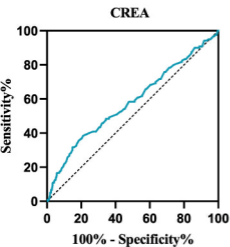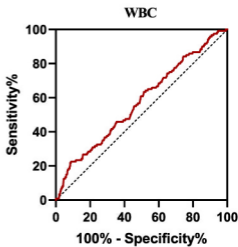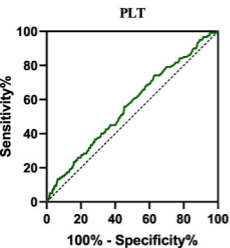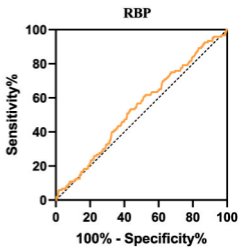

Supplement: Supplementary Materials — Sup Table 1: General linear model analysis of potential risk factors for PAD. Abbreviations: PAD: peripheral artery disease; CysC: cystatin C; HBP: hypertension; PLT: platelets. Sup Table 2: multiple linear regression analysis and collinearity diagnostics of potential risk factors for PAD and ABI measurements. Abbreviations: PAD: peripheral artery disease; ABI: ankle-brachial index; RBC: red blood cell; WBC: white blood cell; PLT: platelets; CysC: cystatin C. Sup Figure 1: flowchart of the study. Sup Figure 2: ROC curve of other potential risk factors to predict PAD. Abbreviations: ROC: receiver operating characteristic; PAD: peripheral artery disease; RBC: red blood cell; uCREA: urine creatinine; uMALB: urine microalbumin; BUN: urea nitrogen; CREA: creatinine; WBC: white blood cell; PLT: platelets; RBP: retinol binding protein. [file 5064264.f1.zip › Sup fig2 (1).pdf]
